# Monolithic Stacked Dielectric Elastomer Actuators

**DOI:** 10.3389/frobt.2021.714332

**Published:** 2021-11-25

**Authors:** Jun Shintake, Daiki Ichige, Ryo Kanno, Toshiaki Nagai, Keita Shimizu

**Affiliations:** Department of Mechanical and Intelligent Systems Engineering, School of Informatics and Engineering, The University of Electro-Communications, Chofu, Japan

**Keywords:** dielectric elastomer actuators, soft robotics, molding, microfluidics, 3D printing

## Abstract

Dielectric elastomer actuators (DEAs) are a promising actuator technology for soft robotics. As a configuration of this technology, stacked DEAs afford a muscle-like contraction that is useful to build soft robotic systems. In stacked DEAs, dielectric and electrode layers are alternately stacked. Thus, often a dedicated setup with complicated processes or sometimes laborious manual stacking of the layers is required to fabricate stacked actuators. In this study, we propose a method to monolithically fabricate stacked DEAs without alternately stacking the dielectric and electrode layers. In this method, the actuators are fabricated mainly through two steps: 1) molding of an elastomeric matrix containing free-form microfluidic channels and 2) injection of a liquid conductive material that acts as an electrode. The feasibility of our method is investigated via the fabrication and characterization of simple monolithic DEAs with multiple electrodes (2, 4, and 10). The fabricated actuators are characterized in terms of actuation stroke, output force, and frequency response. In the actuators, polydimethylsiloxane (PDMS) and eutectic gallium–indium (EGaIn) are used for the elastomeric matrix and electrode material, respectively. Microfluidic channels are realized by dissolving a three-dimensional printed part suspended in the elastomeric structure. The experimental results show the successful implementation of the proposed method and the good agreement between the measured data and theoretical predication, validating the feasibility of the proposed method.

## Introduction

Dielectric elastomer actuators (DEAs) are one of the soft actuator technologies commonly used in the soft robotics fields (Brochu and Pei, 2010; Anderson et al., 2012; Romasanta et al., 2015; Rosset and Shea, 2016; Gu et al., 2017; Hines et al., 2017; Rich et al., 2018; Shintake et al., 2018; Gupta et al., 2019) due to their excellent characteristics: large actuation strains [e.g., areal strain more than 1,000% (Li et al., 2013)], fast responses [e.g., actuation at 600 Hz (Ji et al., 2019)], and theoretically high electromechanical efficiency of up to 90% (Brochu and Pei, 2010). Simple structure and electrically driven nature of DEAs enable the fabrication of devices of various sizes, ranging from millimeter to meter scale (Rosset and Shea, 2016; Gu et al., 2017).

A typical DEA comprises an elastomeric membrane sandwiched between two stretchable electrodes. When subjected to a high voltage potential, electrical charges induced in the electrodes attract each other and generate an electrostatic force called Maxwell stress. Subsequently, the membrane shrinks in the thickness direction and expands in the planar directions. Moreover, elastomeric membranes used in DEAs are usually thin, ranging from 1 µm [a silicone elastomer, reported in ref (Ji et al., 2018)] to 1,000 µm [acrylic elastomer, VHB4910 (M Coporation., 2018)]. Therefore, multiple sets of the membranes and electrodes are stacked to yield useful actuation strokes in the thickness direction, like a muscle. Previous studies have presented various methods for fabricating stacked DEAs where the dielectric and electrode layers are alternately staked (Kovacs et al., 2009; Matysek et al., 2011; Ji et al., 2019), blade-casted (Li et al., 2018), spin coated (Lotz et al., 2011; Duduta et al., 2019), printed (Araromi et al., 2011; Reitelshöfer et al., 2016; McCoul et al., 2017; Chortos et al., 2020), or folded together (Carpi et al., 2007; Nguyen et al., 2014). However, these fabrication methods often require a dedicated setup with complicated processes or sometimes require laborious manual stacking of the layers, which may increase the failure rate of the actuators.

Herein, we propose a method to monolithically fabricate stacked DEA without alternately stacking the dielectric and electrode layers. In this method, stacked DEAs are mainly fabricated through two steps: 1) molding of an elastomeric matrix containing free-form microfluidic channels and 2) injection of liquid conductive material. The microfluidic channels possess the shape of stacked electrodes, which are formed by dissolving an inner part suspended in an elastomeric matrix. Owing to the use of molding process, the shapes of both the electrodes and elastomer structure can potentially be tailored by the geometry of the molded parts. Therefore, the proposed method is expected to provide high design flexibility for the creation of stacked DEA in various forms. Additionally, the relatively simple process may contribute to minimizing the failure rate of the actuators and the required fabrication setup. In this study, we investigate the feasibility of our method by fabricating and characterizing monolithic DEAs with multiple electrodes (2, 4, and 10). The fabricated actuators are characterized in terms of actuation stroke, output force, and frequency response.

## Methods

**FIGURE 1:**
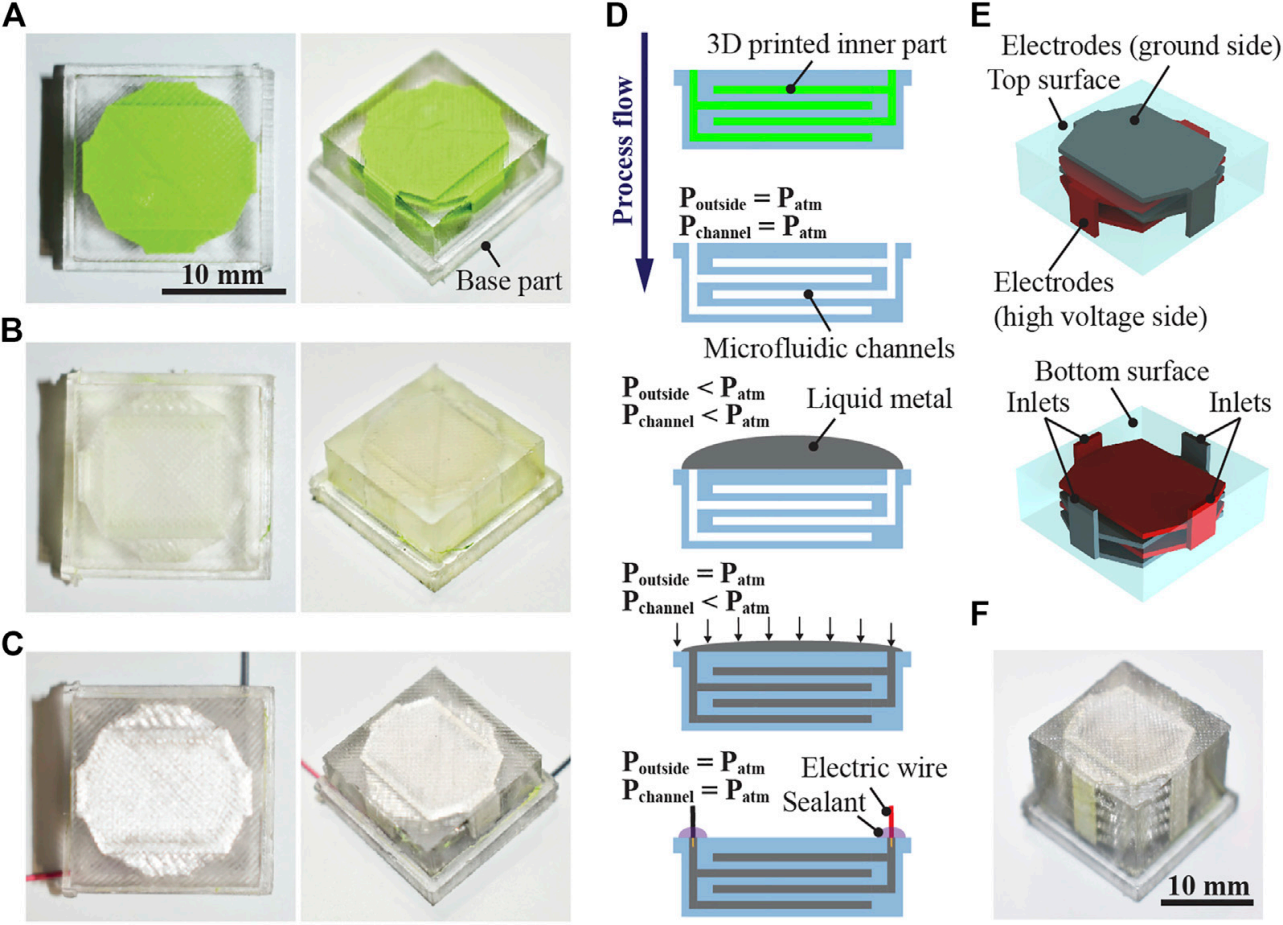
Monolithic stacked DEAs fabricated in this study, comprising a silicone elastomer matrix and liquid metal. **(A)** Elastomeric matrix with a 3D printed part that has an electrode shape. **(B)** Elastomeric matrix after dissolution of the inner part, wherein empty channels remain. **(C)** Elastomeric matrix after injection of the liquid metal into the empty channels and completion of the wiring. **(D)** Process flow of the fabrication. The 3D printed inner part is dissolved, which becomes an empty channel that has an electrode shape. The liquid metal is then injected into the microfluidic channels by the pressure difference between the channel and the outside of the elastomeric matrix. **(E)** Structural view of the actuator. The electrodes are aligned such that opposing polarity alternately appears when subjected to the applied voltage. **(F)** Monolithic stacked DEA with 10 electrodes.

The method proposed herein for fabricating monolithic DEAs combines two technologies employed in the microfluidics field: scaffold removal and vacuum filling. The former is a method used to create empty microfluidic channels by dissolving three-dimensional (3D) printed components suspended in an elastomeric matrix (Saggiomo and Velders, 2015). The latter is a hands-free injection technique where a liquid metal [eutectic gallium–indium, EGaIn (Dickey et al., 2008)] is pushed into microfluidic channels with dead-ends using a suction force because of the pressure difference inside and outside the channels (Lin et al., 2017). Liquid metals are compatible with DEAs when applied to DEAs as electrodes (Liu et al., 2013; Wissman et al., 2014; Pan et al., 2019; Piskarev et al., 2020; Tahidul Haque et al., 2020). Figures 1A–C depict images taken at different stages of the fabrication process of a monolithic DEA containing four electrodes, and Figure 1D represents the process flow. As shown in Figure 1E, the microfluidic channels are aligned such that opposing polarities face each other across an elastomer domain that acts as a dielectric layer. Subsequently, the entire structure functions similar to stacked DEAs. Figure 1F displays a monolithic DEA with 10 electrodes fabricated through the same process. Extension of both the inner part and mold allows the realization of an actuator with a high number of electrodes.

### Fabrication Process

As previously mentioned, the fabrication process of the monolithic DEAs comprises the molding of an elastomeric structure with free-shaped channels and the injection of liquid metal. First, the electrode part and mold were fabricated using an FDM-type 3D printer (Ultimaker, Ultimaker three Extended) with CAD software (Dassault Systèmes, SolidWorks). Polylactic acid (PLA) was employed as the material for the 3D printed parts while polyvinyl alcohol (PVA) was used as a water-soluble support material. After dissolving the PVA support structures, the 3D printed part consisting of an electrode shape and microfluidic channels was placed in the mold. Polydimethylsiloxane (PDMS, Dow Corning, Sylgard 184) prepared with the manufacturer-recommended ratio (crosslinking agent to base polymer ratio of 1:10) was poured into the assembled mold, which was then placed in a vacuum chamber for 30 min to remove residual air bubbles. Thereafter, the sample was cured at 40°C for 12 h in an oven. The subsequent removal of the mold afforded an elastomeric matrix with the 3D printed electrode part suspended inside (Figure 1A).

To dissolve the electrode part, the elastomeric matrix was immersed in a solvent (95% dichloromethane). In a preliminary test, we found that dichloromethane well dissolved the 3D printed part composed of PLA; in contrast, the part did not dissolve when other solvents such as acetone and isopropanol were used. In case of using acetone and isopropanol, probably the presence of additives in the PLA filament provided by the manufacturer of the 3D printer prevented complete dissolution. Furthermore, we experimentally confirmed that the dissolution time depends on the number of electrodes. In this study, monolithic DEAs with 2, 4, and 10 electrodes were fabricated and the corresponding dissolution times were 24, 48, and 144 h, respectively. These times are not the minimum values but are the times sufficient for dissolving the internal parts. After the dissolution, the sample was dried in a fume hood at room temperature for 24 h, yielding an elastomeric matrix with an empty channel (Figure 1B).

Next, a liquid metal, EGaIn (75% gallium and 25% indium), was injected according to the process shown in Figure 1D. The liquid metal was placed on the inlet of the channel in the elastomeric matrix. The entire sample was placed in a vacuum chamber, decompressed for 15 min, and then released to atmospheric pressure. In these steps, the liquid metal flowed into the channel due to the pressure difference between the channel and the outside of the structure; it completely filled the empty domains and formed electrodes inside the elastomeric structure. After placing electric wires and sealing, the monolithic DEA was finally fabricated (Figure 1C). The fabricated actuators had the following design dimensions: the cross-sectional area was 15 × 15 mm, the overlap area of the electrodes was 10 × 10 mm, and the total heights of the devices with 2, 4, and 10 electrodes were 2.5, 4.5, and 10.5 mm (excluding the base part indicated in Figure 1A which has the thickness of 2 mm), respectively. The thickness of the dielectric layers (i.e., the gap between the electrodes) was set to 500 µm. In addition, the thickness of the elastomer layer above the top electrode and below the bottom electrode was also set to 500 µm. The dimensional accuracy and uniformity of the electrode layers depend on the printing resolution of the 3D printer. Since the dimensional accuracy of the 3D printer used in this study (Ultimaker, Ultimaker three Extended) is 50 ± 5 µm (Msallem et al., 2020), we expect that the dimensional accuracy of our actuators is within a similar range. It should be noted that the trueness of the printer was also reported to be 160 ± 9 µm. The uniformity of the electrodes is likely more important with larger surface areas, which in turn may require sophisticated calibration of the printer and optimization of the support structure geometry. Environmental factors such as temperature and humidity must also be considered in such printing. In this study, 3D printing was performed at room temperature (∼24°C) and humidity (∼60% relative humidity).

### Actuator Model

The actuation of DEA is caused by the Maxwell stress squeezing the dielectric layer between the electrodes. This electrostatic stress can be expressed as (Pelrine et al., 1998; Carpi et al., 2008)

$$\sigma_{\text{M}}=\varepsilon_{0}\varepsilon_{\text{r}}E^{2}$$ (1)

where $\varepsilon_{0}$ is the permittivity of free space, $\varepsilon_{\text{r}}$ the relative permittivity of the elastomeric matrix, and E is the electric field between the electrodes ( E=V/d , where V is the applied voltage and d is the thickness of dielectric). To express the deformation of the dielectric layer in response to the Maxwell stress, we employ the Yeoh hyperelastic material model (Yeoh, 1993). This model takes the form of the strain energy density function as

$$W=\sum_{i=1}^{3}C_{i}{\left(I_{1}-3\right)}^{i}$$ (2)

where $C_{i}$ is the material constant and $I_{1}$ is the strain invariant $\left(I_{1}=\lambda_{1}^{2}+\lambda_{2}^{2}+\lambda_{3}^{2}\right)$ . Additionally, $\lambda_{1}$ , $\lambda_{2}$ , and $\lambda_{3}$ are the stretch ratios in the length, width, and thickness directions, respectively. Assuming that the material is incompressible (i.e., $\lambda_{1}\lambda_{2}\lambda_{3}=1$ ) and the planar shape of the actuator is square (i.e., $\lambda_{1}=\;\lambda_{2}$ ), the stress in the thickness direction is expressed as

$$\sigma_{3}=\lambda_{3}\frac{\partial W}{\partial I_{1}}=2\left(\lambda_{3}^{2}-\frac{1}{\lambda_{3}}\right)\sum_{i=1}^{3}iC_{i}{\left(\lambda_{3}^{2}+\frac{2}{\lambda_{3}}-3\right)}^{i-1}$$ (3)

Under the application of voltage, $\sigma_{\text{M}}$ (electrostatic stress) and $\sigma_{3}$ are in a state of equilibrium (Patrick et al., 2007), and the following equation is obtained

$$-\varepsilon_{0}\varepsilon_{r}{\left(\frac{V}{d_{0}\lambda_{3}}\right)}^{2}=2\left(\lambda_{3}^{2}-\frac{1}{\lambda_{3}}\right)\sum_{i=1}^{3}iC_{i}{\left(\lambda_{3}^{2}+\frac{2}{\lambda_{3}}-3\right)}^{i-1}$$ (4)

where $d_{0}$ is the initial thickness of the elastomer layer $\left(d=\;d_{0}\lambda_{3}\right)$ . Solving Eq. 4 gives the stretch ratio in the thickness direction (i.e., the axial direction of the actuator), which is $\lambda_{3}$ as a function of the applied voltage V . The actuation strain is given as

$$\text{S}=\lambda_{3}-1$$ (5)

The amount of actuation stroke is then represented as

α=Sh (6)

where h is the effective height of the actuator calculated from the number of electrodes N and $d_{0}$ , or $h=d_{0}\left(2N-1\right)$ . In this study, N is taken as 2, 4, and 10. The actuation force in the thickness direction can be found by using Eq. 1 and considering the overlap area of the electrodes and the thickness of the elastomer layer d . This gives

$$F=\varepsilon_{0}\varepsilon_{r}\frac{A_{e0}}{\lambda_{3}}{\left(\frac{V}{d_{0}\lambda_{3}}\right)}^{2}$$ (7)

where $A_{e0}$ is the initial value of the overlap area of the electrodes. During the force characterization, explained in *Experimental Procedure*, the top and bottom surfaces of the actuator are fixed, which may limit the actuated deformation of the structure in the planar directions. In such cases, Eq. 7 is no longer valid as it considers the areal expansion of the electrodes and dielectrics. Instead, when the device is considered as a capacitor with rigid parallel electrodes, the force is given as follows:

$$F=\frac{1}{2}\varepsilon_{0}\varepsilon_{r}A_{e0}{\left(\frac{V}{d_{0}}\right)}^{2}$$ (8)

We compare the theoretical values calculated using Eqs 6, 8 with the experimental data. The results are discussed in *Results*.

### Experimental Procedure

#### Characterization of Immersion Influence on Mechanical and Material Properties

During the dissolution process of the electrode part, explained in *Fabrication Process*, the elastomeric matrix is immersed in the solvent. This process could affect the chemical composition of the material. In this case, the mechanical and material properties of the elastomer change and afford different actuation characteristics depending on the dissolution time. In the actuator model discussed in the previous section, the parameters that could be affected by the dissolution process are the material constants $C_{i}$ and the relative permittivity $\varepsilon_{r}$ . The material constant $C_{1}$ is related to the Young’s modulus Y by $Y=6C_{1}$ . This means that changes in $C_{i}$ lead to changes in the elastomer’s stiffnesses and stress-strain behaviors. To investigate the influence of the dissolution process on these parameters, tensile test and capacitance measurement were performed on the PDMS membranes immersed in the solvent.

In the tensile test, a 1-mm-thick PDMS membrane fabricated using a mold was punched into a set of samples with dumbbell shapes (JIS K6251, ISO 37, Type 1A). The curing conditions of the membrane were identical to those of the actuator (40°C and 12 h). Moreover, the samples were immersed in the solvent for different times including those used in the fabrication of the actuators: 24, 48, 96, and 144 h. After drying in a fume hood at room temperature for 24 h, all samples were tested in a universal testing machine (Shimadzu, AGS-20NX), and the stress–strain curves were recorded. The samples without solvent immersion were also characterized as controls. These tests were performed at a tensile speed of 60 mm/min until the specimens broke. The values for $C_{i}$ were then obtained by fitting the Yeoh hyperelastic material model. The stress in the length direction (tensile direction) is expressed as

$$\sigma_{1}=\lambda_{1}\frac{\partial W}{\partial I_{1}}=2\left(\lambda_{1}^{2}-\frac{1}{\lambda_{1}}\right)\sum_{i=1}^{3}iC_{i}{\left(\lambda_{1}^{2}+\frac{2}{\lambda_{1}}-3\right)}^{i-1}$$ (9)

where $\lambda_{1}$ , $\lambda_{2}$ , and $\lambda_{3}$ are the stretch ratios in the length, width, and thickness directions, respectively. The material constants $C_{1}$ , $C_{2}$ , and $C_{3}$ were obtained by fitting Eq. 9 to the measured stress-strain curve. Then, the Young’s modulus Y of the sample was acquired to represent the stiffness change in the elastomer.

In the capacitance measurement, a set of 1.5-mm-thick PDMS membranes were prepared using a mold. The curing condition of the membranes was identical to that of the actuator (40°C and 12 h). The membranes were immersed in the solvent for different times including those used in the fabrication of the actuators: 24, 48, 96, and 144 h. After drying in a fume hood at room temperature for 24 h, every membrane was sandwiched between two aluminum plates, forming a capacitor with an electrode area of 20 × 10 mm. The capacitance of the samples was measured using an LCR meter (TEXIO, LCR6000). The relative permittivity $\varepsilon_{\text{r}}$ was then calculated from the relation $\varepsilon_{\text{r}}=C_{m}d/\varepsilon_{0}A$ , where $C_{m}$ is the measured capacitance, d is the gap (i.e., the thickness of membrane), and A is the electrode area. As a control, the samples without solvent immersion were also characterized. During the above-mentioned tests, three samples were characterized, and their average value was reported.

#### Characterization of Monolithic DEAs

The fabricated actuator was vertically fixed on a mechanical stage, above which a laser displacement sensor (OPTEX FA, CDX-L15) was set to measure the displacement change of the actuator in the thickness direction, which was taken as the actuation stroke. The resolution of the laser displacement sensor was 0.01 µm (OPTEX FA CO, 2021). The schematics in Figure 3A represent this measurement method. Furthermore, the actuator was connected to a high-voltage DC/DC converter (CB101, XP Power), where an input low voltage signal was supplied from a function generator (Matsusada, eK-FGJ). A low voltage power supply was used to drive the DC/DC converter. Test voltages from 0 to 10 kV, in increments of 1 kV (corresponding to the electric field of 0–20 V/μm with 2 V/μm increments), were applied to the actuator. With the same setup, the frequency response of the actuator was characterized. The waveform of the input voltage was sinusoidal with frequency ranging from 1 to 10 Hz, in increments of 1 Hz. In this test, the voltage was maintained as 3 kV. To measure the output force, a probe connected to load cell (LSB200, FUTEK) was attached to the free end (i.e., the top surface) of the actuator. The schematics in Figure 3C represent this measurement method. In this experiment, the actuator with 10 electrodes was measured (voltage range: 0–10 kV, in increments of 1 kV), since the displacement yielded by the other actuators with two and four electrodes is insufficient for pushing the load cell probe. During the above-mentioned tests, three samples were characterized, and their average value was reported.

## Results

### Characterization of the Immersion Influence on Mechanical and Material Properties

**FIGURE 2:**
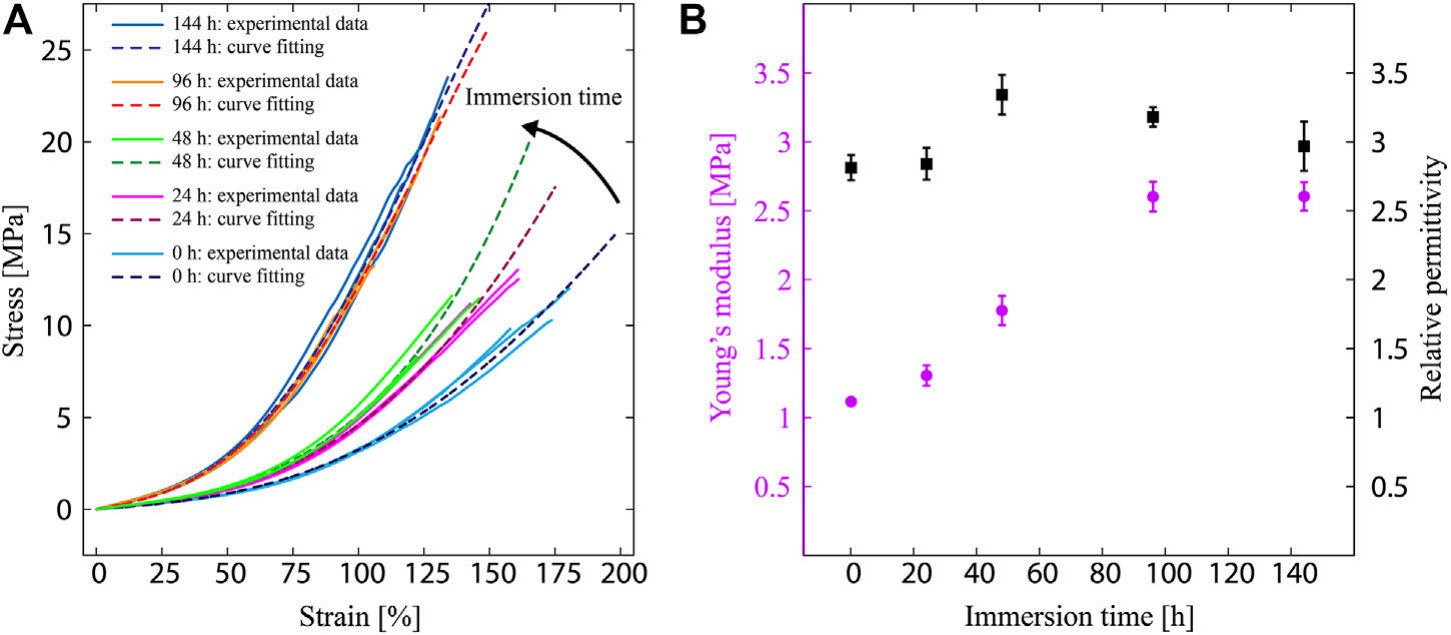
**(A)** Measured stress–strain curve for the PDMS samples under different immersion times. **(B)** Measured Young’s modulus and relative permittivity of PDMS samples as function of the immersion time.

We observed that the stress–strain behavior of PDMS changes with the immersion time, as shown in Figure 2A. The stress–strain curve visibly changes between the immersion times of 0 and 144 h, suggesting that the elastomer becomes stiffer. Moreover, the data shows almost no change between 96 and 144 h, indicating that the change in the stress–strain behavior, and thus the mechanical property of PDMS, saturates at some point between the immersion times of 48 and 96 h. Therefore, as shown in Figure 2B, the Young’s modulus also saturates and takes a value of 2.6 ± 0.1 MPa. Furthermore, the relative permittivity decreases as the immersion time increases and reaches a value of 2.97 ± 0.18 at 144 h.

### Characterization of Monolithic DEAs

**FIGURE 3:**
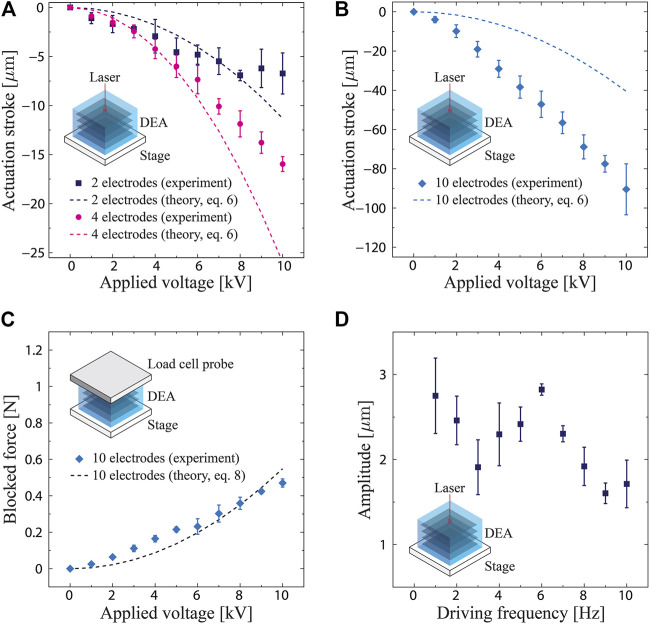
Measured performance of the fabricated actuators. **(A)** Actuation stroke as a function of the applied voltage for the actuators with two and four electrodes. **(B)** Actuation stroke as a function of the applied voltage for the actuator with 10 electrodes. **(C)** Output force as a function of the applied voltage for the actuator with 10 electrodes. **(D)** Amplitude as a function of the driving frequency (voltage 3 kV).

The performance of the monolithic DEAs fabricated herein, characterized in terms of the actuation stroke, output force, and frequency response, is summarized in Figure 3. Note that the active deformation of the actuator is contraction, which has negative values. As expected from Eq. 4, the actuation stroke depicts a quadratic change because of the Maxwell stress squeezing the dielectrics, whose intensity is proportional to square of the electric field. Their magnitude also increases with the number of electrodes. The actuation strokes for the actuators with two and four electrodes have values of −6.7 ± 2.1 µm (−0.4 ± 0.1% strain) and −15.0 ± 0.8 µm (−0.5 ± 0.02% strain), respectively, at 10 kV (corresponding to the electric field of ∼20 V/μm) (Figure 3A). The dashed lines in Figure 3A indicate the theoretical values calculated using Eq. 6 with $\varepsilon_{0}=8.85\times 10^{-12}$ F/m, $d_{0}=0.5$ mm, and the measured $C_{i}$ and $\varepsilon_{r}$ values discussed in the previous section. The model shows that the trends generally agree with the experimental data. The discrepancy between the theoretical and experimental values results from passive elastomeric domains in the actuator that reduce the actuation strokes.

In the case of the actuator with 10 electrodes, the actuation stroke assumes a value of −90.5 ± 13.0 µm (−1.0 ± 0.1% strain) at 10 kV, as shown in Figure 3B. A relatively large difference is observed between the model and experimental data. As for the output force, the actuator with 10 electrodes exhibits 0.47 ± 0.02 N at 10 kV (Figure 3C). The data again show a quadratic change. The theoretical values calculated from Eq. 8 well match the measured data, suggesting that the boundary conditions of the actuator influence the measured force. Figure 3D shows the measured amplitude of the actuator with two electrodes as a function of the driving frequency. The amplitude is largest at 6 Hz, indicating the presence of resonance.

## Discussion

The experimental results demonstrate the successful implementation of the proposed method for fabricating monolithic stacked DEAs, from which the objective of this study—validation of the proposed method—was achieved.

Overall, the theoretical model, which considers the change in mechanical and material properties, well captures the behavior of the actuators, suggesting that the model is effective at designing actuators made using the proposed method. There are several possible explanations for the discrepancies between the theoretical and experimental values. The first is manufacturing error. If the thickness of the dielectric part is small, the electrostatic stress will increase and the actuation will be larger. The second explanation is that the changes in the mechanical and material properties due to the solvent immersion may be nonuniform throughout the structure. In this case, the dielectric part may be softer than that modeled, and thus, the observed actuation strokes will be larger. This effect may be significant, especially for the actuators with 10 electrodes because they have a large volume. The third is a slight reduction in the actuator volume due to the extraction of uncross-liked material during the immersion in the solvent (Glover et al., 2020). This may make the dielectric layers slightly thinner and, in turn, the actuation strokes larger due to an increase in the electrostatic stress. Lastly, while these three scenarios would increase the actuation strokes, the presence of passive elastomeric domains in the actuators would reduce the strokes. Therefore, the measured data likely represents a combination of these factors. Regarding the experimental results, the resonance observed in the frequency response characterization suggests that the actuation stroke can be magnified and controlled by matching the driving frequency. This characteristic may be particularly useful for devices that perform periodic motions, such as mobile robots and vibrating devices.

The actuation strain values observed for the actuators fabricated in this study range from 0.4 to 1.0% in an electric field of ∼20 V/μm. These numbers are noticeably small compared to those of stacked DEAs fabricated by other methods: 46% at ∼50 V/μm (Kovacs et al., 2009), 24% at 150 V/μm (Duduta et al., 2019), 15.5% at ∼12 V/μm (Carpi et al., 2007), 9% at 25 V/μm (Chortos et al., 2020), and 4.5% at ∼13 V/μm (Li et al., 2018). There are two main reasons behind this. The first is the fact that the electric field applied to our actuators is low (∼20 V/μm). The electric field is limited by the experimental equipment, which allows applied voltages of up to 10 kV. Applying higher electric fields below the breakdown strength of the elastomer leads to larger actuation strains. To produce this effect, one could simply use higher voltage or reduce the thickness of the dielectric layers. In the latter case, the thickness of the dielectric layers depends on the minimum allowable 3D-printed layer thickness for the scaffold. This minimum thickness depends on the performance and type of 3D printer, but a value around 100 μm can be reasonably expected given that the printer used in this study has a minimum layer height of 50 μm (Stano et al., 2020) and a dimensional accuracy of 50 μm (Msallem et al., 2020). However, as the performance of 3D printers continues to improve, the achievable minimum dielectric layer thickness in our method is expected to become smaller. The second reason for the low actuation strains of our actuators is that the Young’s modulus of dielectric layers (1.1–2.6 MPa) is significantly higher than that used in other studies. For example, Ecoflex 00-20 (Smooth-On), which was used by Li et al. (2018), has a Young’s modulus is ∼0.05 MPa (Jin et al., 2021). Within the same magnitude of electrostatic stress, the strain is greater for softer elastomers. Therefore, by identifying and incorporating softer elastomers that are compatible with our method, the actuation strains of our actuators can be expected to become larger. Candidates for such elastomers include the Ecoflex series described above and Sylgard 184 with a modified mixing ratio (Glover et al., 2020). It should be noted that this work will also require the exploration of suitable solvents and the investigation of their influence on the mechanical, material, and electrical properties of chosen elastomers.

Since this study aims to validate the feasibility of the proposed method comprising 1) molding of an elastomeric matrix containing free-form microfluidic channels and 2) the injection of a liquid conductive material, the shape of the tested actuators is limited to the simplest form. However, owing to the simple molding process and the use of 3D printed parts, our method provides high design flexibility; thus, it can be potentially applied to stacked DEAs of diverse forms. These actuator shapes include cylinder, triangle pole, ellipsoid, and other 3D free forms. In future studies, we will investigate the design flexibility of our novel method and the applicable materials as well as the extension of the model to predict the characteristics of actuators for a given specification. To assure prediction accuracy, the model will also consider the presence of passive elastomeric domains in the actuators as well as the volume reduction by immersion in the solvent. Related to this, the fabrication parameters should be optimized in order to reduce the non-uniformity of the mechanical and material properties within the actuator structure. In parallel, we will clarify how far the actuators’ performance can be improved, while considering the resolution of the mold and suitable materials. Lifetime and reliability are particularly important aspects for evaluating actuation performance. With the aid of future work mentioned above, our method is expected to derive DEA-based soft robots and devices of various forms in diverse applications.

## Data Availability

The raw data supporting the conclusion of this article will be made available by the authors, without undue reservation.
